# Hepatocellular Carcinoma (HCC), Where do we stand? Current situation

**DOI:** 10.12669/pjms.36.3.1594

**Published:** 2020

**Authors:** Muhammad Hafeez, Muhammad Nadeem, Mahmood Ahmed, Faheem-ur-Rehman

**Affiliations:** 1 Dr. Muhammad Hafeez, FCPS (Med), FCPS (Gastroenterology). Department of Medicine, Combined Military Hospital (CMH), Multan, Pakistan; 2 Dr. Muhammad Nadeem, FCPS (Med), FCPS (Med Onc). Department of Medicine, Combined Military Hospital (CMH), Multan, Pakistan; 3 Dr. Mahmood Ahmed, FCPS (Rehab Med), MSc (Pain Medicine). Department of Medicine, Combined Military Hospital (CMH), Multan, Pakistan; 4 Dr. Faheem-ur-Rehman, FCPS (Med). Department of Medicine, Combined Military Hospital (CMH), Bahawalpur, Pakistan

**Keywords:** Hepatocellular Carcinoma, Staging of HCC, BCLC, MELD

## Abstract

**Objective:**

To identify the stage of Hepatocellular Carcinoma (HCC) at the time of presentation.

**Methods:**

This cross sectional observational prospective study was carried out at Gastro Department of Combined Military Hospital (CMH) Multan from August 2017 to December 2018. Patients were diagnosed on the basis of alpha fetoprotein, abdominal ultrasound, triphasic contrast enhanced computerized tomography (CECT). They were evaluated for etiology including Hepatitis B, C and non B & C. The patients were inquired about the previous treatment and when they came to know about the HCC. Staging of the tumor was done on the basis BCLC (Barcelona cancer liver clinic) and Melan’s criteria. Performance status (PS) of the patient was checked by Eastern Cooperative Oncology Group (ECOG) criteria. Severity of cirrhosis was assessed by CTP (Child Turcotte Pugh) and Model for end stage liver disease (MELD) score. The data was analyzed in IBM SPSS version 22.

**Results:**

Out of 135 patients 78% were males and 22% females. Age Mean SD was 58.81± 9.366. Frequency of hepatitis C, B, combined B, C and non-B non-C was 80%, 11%, 2.8% and 6.2% respectively. 96(73.8%) never got the treatment before for Hepatitis. 81(62.3%) came to know first time on this index admission. Maximum numbers of patients were in BCLC stage B i.e. 82(55.2%) with ECOG grade of one i.e.57 (39.3%), at the time of presentation. Mean MELD and CTP score were 12.24, 7.34 (class B) respectively.

**Conclusion:**

HCV was the most common in HCC, never treated before, presented for the first time in advance stage of the disease where very limited treatment options left behind.

## INTRODUCTION

Hepatocellular carcinoma was the second most common cancer death in males and 7^th^ amongst females in 2012 worldwide.^1^-^3^ In Pakistan frequency of hepatobiliary malignancy in males is 10.7% of all cancers.^4^ Prevalence of HCC in Pakistan varies 3.5-16% of all malignancies and Chronic Hepatitis B, C and D related cirrhosis are major causes. In two different studies frequency of HCC in Pakistan secondary to HCV is 87%, 66% and in HBV 22% and 43% has been reported.^5^ This deadly complication can be prevented by putting the patients at risk in regular surveillance programme.^6^ According to American association for the study of liver disease (AASLD) guidelines any mass in Chronic Liver Disease (CLD) patients is likely HCC.^7^ AASLD and European Association for the study of liver (EASL) disease recommends six monthly Ultrasound liver and serum AFP to avoid missing fresh cases.^8^ In this era of high public awareness and social media still our patients are ignorant about the serious consequences of hepatitis. Historically patients present late despite being symptomatic and impairment of liver functions. According to a report, majority of patients presents when very limited treatment options of cure and less than 1% chances of resection left.^8^ Less than 10% HCC patients are diagnosed with screening in Pakistan.^9^,^10^ This fact has been pointed out in a number of studies but there are very few studies that exactly quantify this issue, that’s why this study was done to identify the stage at presentation of HCC in our population in order to increase awareness among health professionals to be vigilant to identify HCC earlier so that curative options of treatment can be exercised.

## METHODS

This cross sectional was carried out at CMH Multan from August 2017 to April 2018 with ethical approval by ethical committee of the Hospital. (August 21, 2019) A total of 135 patients with diagnosis of Hepatocellular Carcinoma were included using nonprobability convenience sampling technique after informed consent from the patients.

Study population comprised of patients presenting in CMH Multan, a tertiary care hospital, for follow up of DCLD, had SOL (space occupying lesion) confirmed HCC after investigations mentioned below were included. Patients having SOL liver other than HCC, with incomplete records and on treatment, not alive or unwilling to be part of study were excluded.

Demographic data including age, and gender were noted. Basic investigation including complete blood counts, liver functions including serum albumin, INR and renal function tests were done. Serology for Hepatitis B, C and D was done. Autoimmune profile including antinuclear antibody (ANA), anti-smooth muscle, anti-liver kidney and anti-mitochondrial antibodies were done to find out etiology. Serum ferritin, cerulo-plasmin, 24 urinary copper levels wherever required were carried out to rule out hemochromatosis and Wilson’s disease. Abdominal ultrasound was performed. Space occupying lesions in the background of chronic liver disease were further investigated for HCC that included serum α fetoprotein and Tri-phasic CECT abdomen.

After establishing the diagnosis staging was done by BCLC and Melan’s criteria.^11^ It is based on tumor size, its spread, patients’ performance status assessed by ECOG criteria.^12^ Severity of the underlying chronic liver disease was assessed by Child Pugh^13^,^14^ and MELD^15^ criteria.

Patients with Child Pugh score of 5-6 were classified as Child Class A, those with 7-9 were classified as Child Class B while those with Child Pugh score of 10-15 were classified as Child Class C. MELD score was calculated according to following formula:

MELD = 3.8[Ln serum bilirubin (mg/dL)] + 11.2[Ln INR] + 9.6[Ln serum creatinine (mg/dL)] +6.4, where Ln is the natural logarithm

Data was entered in SPSS v 22.0. Frequencies of patients according to gender, hepatitis status, treatment status, time since diagnosis of hepatocellular carcinoma, child class, BCLC staging and ECOG performance status were determined. Descriptive statistics were applied. Means and Standard Deviations were determined for Age, Serum Total Bilirubin, Albumin, INR, Child Score, Serum Creatinine Levels, and Total Leukocyte Count, Hemoglobin levels (Hb), Platelet Count, Alpha Fetoprotein, ALT, ALK and MELD score.

## RESULTS

Patient characteristics like gender, status of hepatitis, previous history of hepatitis treatment, time since diagnosis of HCC, Child Class, BCLC Stage and ECOG Performance status of 135 included patients have been displayed in Table-I.

**Table-I:** Profile of Patients included in present study.

Gender	Frequency	Percentage
Male	106	78.5
Female	29	21.5
***Hepatitis Status***		
Hepatitis C	109	80.7
Hepatitis B&C	4	3.0
Hepatitis B	14	10.4
Non B&C	4	3.0
Cryptogenic	4	3.0
***Anti-viral treatment for chronic hepatitis***		
Received	37	27.4
Not received	98	72.6
Time since diagnosis of HCC	85	63.0
<1 month	18	13.3
1-3 months	13	9.6
3-6 months	6	4.4
6-12 months	7	5.2
12-18 months	1	0.7
2-2.5 years	3	2.2
>2.5 years	2	1.5
***Child Class***		
A	49	36.3
B	63	46.7
C	23	17.0
***BCLC Stage***		
Stage 0	4	3.0
Stage A	16	11.9
Stage B	78	57.8
Stage C	34	25.2
Stage D	3	2.2
***ECOG Performance Status***		
Fully active	21	15.6
Restricted physical activity, able to do sedentary work	55	40.7
Ambulatory capable of self-care unable to carry out any work activities	30	22.2
Self-limited care on chair more than 50% working hours	26	19.3
Completely disable cannot carry on any self-care	3	2.2

*Parameters*	*Mean*	*Standard Deviation*

Age of the patient	59.05	9.02
Bilirubin (umol/L)	1.47	0.74
Albumin (g/L)	1.93	0.68
INR	1.47	1.40
Child Score	7.37	2.13
Serum Creatinine umol/l	99.70	70.67
TLC	7.53	3.16
Hb of the patient g/dl	10.71	2.33
Platelet count	159.54	93.95
Alpha Fetoprotein	2169.14	7914.81
ALT	79.62	61.41
ALK	318.37	163.25
MELD Score	12.48	5.92

Patient Characteristics as per BCLC Staging have been shown in Table-II and III. It was observed that most of patients belonged to stage B irrespective of Gender, Status of Hepatitis, treatment Status, duration since diagnosis and ECOG performance status. 103 (85%) of the patients were diagnosed to have HCC recently or on index admission.

**Table-II:** Patient Characteristics as per BCLC Staging.

		BCLC staging				

		Stage 0	Stage A	Stage B	Stage C	Stage D

		Count	Count	Count	Count	Count
Sex of the patient	Male	4	14	56	29	3
	Female	0	2	22	5	0
Hepatitis status	Hepatitis C	4	14	64	25	2
	Hepatitis B&C	0	0	1	3	0
	Hepatitis B	0	1	8	4	1
	Non B&C	0	0	2	2	0
	Cryptogenic	0	1	3	0	0
Treatment for the disease before	Yes	1	11	15	9	1
	No	3	5	63	25	2
Diagnosis HCC since	Diagnosis on admission	4	10	45	24	2
	<1 month	0	3	13	2	0
	1-3 months	0	1	7	4	1
	3-6 months	0	1	4	1	0
	6-12 months	0	0	6	1	0
	12-18 months	0	0	1	0	0
	18-24	0	0	0	0	0
	2-2.5 years	0	0	2	1	0
	>2.5 years	0	1	0	1	0
ECOG Performance status	Fully active	2	4	11	4	0
	Restricted physical activity, able to do sedentary work	0	6	38	11	0
	Ambulatory capable of self-care unable to carry out any work activities	0	4	17	9	0
	self-limited care on chair more than 50% working hours	2	2	12	9	1
	completely disable cannot carry on any self-care	0	0	0	1	2
	Dead	0	0	0	0	0

**Table-III:** Patient Profile as per BCLC staging.

	BCLC staging				

	Stage 0	Stage A	Stage B	Stage C	Stage D
TLC	7.68	6.28	7.60	7.66	10.87
Hb	11.00	10.41	10.69	11.21	6.87
Platelet count	182.00	107.19	165.40	169.21	147.33
Serum Creatinine	99.27	82.42	100.98	97.01	189.67
ALT	74.50	60.94	86.18	72.50	96.67
ALK	362.00	262.69	318.38	343.71	269.67
Billirubin	1.25	1.44	1.38	1.59	3.00
Albumin	1.50	2.13	1.87	2.03	2.00
INR	1.00	2.44	1.34	1.32	2.33
child score	5.75	7.31	7.14	7.76	11.67
AFP	324.85	68.01	2028.21	3877.34	139.30

Above table points 98(72%) HCC patients never got any sort of antiviral therapy before that indicates a big gap between general population and health providers.

## DISCUSSION

Incidence of HCC is more in men especially in HCV prevalent areas of United States,^16^ same is the case in our study i.e. majority are males with HCV between ages of 55 to 64 years. Age-specific rates of men in high-risk African populations (e.g. Gambia and Mali) peak in the 60 to 65 age group and women peak between 65 and 70 years old. These variations of age-specific patterns are most likely related to the differences in the dominant hepatitis virus in the population, the age at viral infection as well as the existence of other risk factors. In India Hepatitis B followed by C was most common cause of HCC.^17^ For the better patient’s survival and to detect new HCC cases early, surveillance of patients with chronic Liver disease has been over emphasized in a number of studies and guidelines^7^,^18^-^20^, but unfortunately 73.8% of our patients were totally unaware about their hepatitis status, didn’t get any specific treatment before and 64% came to know about HCC on the index admission for the first time. This indicates a big gap in communication between health care providers and the patients. Prognosis and treatment plan is assessed by most widely accepted BCLC staging system. According to it in stage A, hepatic resection, liver transplantation is possible, in stage B patients are asymptomatic and have multinodular tumor and in Stage C along with previous features, patient has vessel invasion or extrahepatic spread. Only palliative treatment is possible at Stage B & C.^10^,^21^ Situation is better in India where about half of the patients had BCLC stage A and B in whom definitive therapy could be offered and 65% (688/1062) of HCC were due to HBV and HCV.^22^ One hundred and twenty-one (83.44%) our patients were in BCLC stage B&C with performance status (ECOG) one and above and average Child score seven, when nothing can be done much except palliative treatment. This advanced stage diagnosis is quite high as compared to average global HCC presentation at palliation stage is 25-75%.^23^,^24^ The most common BCLC stage at diagnosis was stage C in North America, Europe, South Korea and China, and stage A in Japan and Taiwan. In Japan and Taiwan, approximately 70% of patients were diagnosed with HCC at BCLC stage 0 or A, and less than 20% were diagnosed at BCLC stage C or D.^25^ Above studies points better surveillance programme in Japan and Taiwan than North America and Europe. Further its need to stress more on implementation of the guidelines rather making more strict surveillance programs. Out of the two scoring models currently being used to assess the severity and prognosis of CLD patients i.e. CTP and MELD.^12^-^14^ MELD is preferred over CTP because of the subjectivity in the later. MELD has been adapted by United Network Organ sharing (UNOS) to prioritize the Cadaveric organ donation of Liver. MELD now is also in consideration to be adopted in different world areas and countries. MELD score assigned specific score in HCC patients depending upon the tumor burden and three months survival renders more proportion of organ donations in this group.^26^ Reason for assigning additional scores are, HCC patients may not demonstrate the degree of hepatic synthetic dysfunction necessary to give them a competitive MELD score, secondly in absence additional points may make them unfit for surgery after three months. In our study BCLC stage B, PS one and above, average CTP score of seven, class B, MELD 12 comes under the heading of more tumor burden and advanced disease and unfortunately don’t merit the priority for transplantation. Only options left are trance arterial chemo embolization (TACE) or tyrosine kinase inhibitors drugs like Sorefenib.

## CONCLUSION

HCC is a sinister complication of Chronic Liver Disease but majority of our patients are diagnosed at advanced stage of the disease with high mortality and limited treatment options.

### Authors’ Contribution

**MH** conceived, designed and did statistical analysis & editing and proof reading of manuscript, is responsible for integrity of research. **MN** helped in collecting data, analyzing, editing and final proof reading. **MA** helped in collecting data, writing, editing and final proof reading of manuscript. **FR** helped in writing, editing and final proof reading of manuscript.
